# Supplementary material for: Are Loneliness and Social Isolation Associated with Quality of Life in Older Adults? Insights from Northern and Southern Europe
Source: Int J Environ Res Public Health. 2020 Nov 20;17(22):8637. doi: 10.3390/ijerph17228637 (PMC7699832; doi:10.3390/ijerph17228637)
Supplement: Supplementary file 1 [file ijerph-17-08637-s001.pdf]

Supplementary Material: Are loneliness and social isolation associated with quality of life in older adults? Insights from Northern and Southern Europe

Giorgi Beridze[1], Alba Ayala[2,3], Oscar Ribeiro[4], Gloria Fernández-Mayoralas[5], Carmen Rodríguez-Blázquez[6,7], Vicente Rodríguez-Rodríguez[5], Fermina Rojo-Pérez[5], Maria João Forjaz[3,6], Amaia Calderón-Larrañaga[1,3]

1. Aging Research Center, Department of Neurobiology, Care Sciences and Society, Karolinska Institutet-Stockholm University, Stockholm, Sweden
2. National School of Public Health, Carlos III Institute of Health, Madrid, Spain
3. Health Services Research on Chronic Patients Network (REDISSEC), Carlos III Institute of Health, Madrid, Spain
4. Center for Health Technology and Services Research (CINTESIS), Department of Education and Psychology, University of Aveiro, Aveiro, Portugal
5. Institute of Economics, Geography and Demography (IEGD), Spanish National Research Council (CSIC), Madrid, Spain
6. National Centre of Epidemiology, Carlos III Institute of Health, Madrid, Spain
7. The Network Center for Biomedical Research in Neurodegenerative Diseases (CIBERNED), Madrid, Spain

* Correspondence: Giorgi Beridze, Aging Research Center, Karolinska Institutet, Tomtebodavägen 18 A, floor 10, 171 65 Solna, Sweden. Email: giorgi.beridze@ki.se

Supplementary table 1. Characteristics of non-respondents in Sweden

	**Participants in wave 6 only** N=881	**Participants in waves 6 & 7** N=2,995	**p-value**
**Age (mean, SD)**	71.5 (10.5)	70.4 (8.7)	.053
50-64	246 (27.9%)	805 (26.9%)	
65-74	312 (35.5%)	1,272 (42.5%)	
75-84	201 (22.8%)	726 (24.2%)	
85+	122 (13.8%)	192 (6.4%)	
**Gender**			.160
Male	404 (45.9%)	1,377 (46.0%)	
Female	477 (54.1%)	1,618 (54.0%)	
**Education level**			<.001
Primary	244 (27.7%)	598 (20.0%)	
Secondary	411 (46.7%)	1,369 (45.7%)	
Tertiary	226 (25.7%)	967 (32.3%)	
Missing	0 (0.0%)	61 (2.0%)	
**Activity limitations (mean, SD)**	1.0 (2.5)	0.4 (1.4)	<.001
0	648 (73.6%)	2,516 (84.0%)	
1+	227 (25.7%)	475 (15.9%)	
Missing	6 (0.7%)	4 (0.1%)	
**Chronic diseases (mean, SD)**	1.6 (1.5)	1.4 (1.4)	<.001
0	215 (24.4%)	878 (29.3%)	
1	272 (30.9%)	932 (31.1%)	
2+	394 (44.7%)	1,180 (39.4%)	
Missing	0 (0.0%)	5 (0.2%)	
**EURO-D (mean, SD)**	2.2 (2.0)	1.9 (1.8)	<.001
<4	643 (73.0%)	2,438 (81.4%)	
≥4	183 (20.8%)	503 (16.8%)	
Missing	55 (6.2%)	54 (1.8%)	
**Memory test**			<.001
Fair/Poor	283 (32.1%)	771 (25.7%)	
Excellent/Very good/Good	598 (67.9%)	2,147 (71.7%)	
Missing	0 (0.0%)	77 (2.6%)	
**Hearing test**			<.001
Fair/Poor	182 (20.7%)	556 (18.6%)	
Excellent/Very good/Good	699 (79.3%)	2,435 (81.3%)	
Missing	0 (0.0%)	4 (0.1%)	
**Eyesight**			<.001
Fair/Poor	145 (16.5%)	379 (12.7%)	
Excellent/Very good/Good	736 (83.5%)	2,612 (87.2%)	
Missing	0 (0.0%)	4 (0.1%)	
**Loneliness (mean, SD)**	3.8 (1.2)	3.7 (1.1)	.110
Low	650 (73.7%)	2,454 (81.9%)	
High	189 (21.5%)	512 (17.1%)	
Missing	42 (4.8%)	29 (1.0%)	
**Social isolation (mean, SD)**	2.0 (0.9)	1.8 (0.9)	<.001
Low	604 (68.6%)	2,241 (74.8%)	
High	210 (23.7%)	588 (19.7%)	
Missing	67 (6.7%)	166 (5.5%)	
**Baseline CASP (mean, SD)**	39.1 (5.2)	39.6 (5.0)	<.001
Missing	71 (8.0%)	124 (4.1%)	

Note: p-values obtained using ANOVA and Pearson’s chi-square test

Supplementary table 2. Characteristics of non-respondents in Spain

	**Participants in wave 6 only** N=1,398	**Participants in waves 6 & 7** N=4,154	**p-value**
**Age (mean, SD)**	71.7 (11.3)	69.9 (10.2)	<.001
50-64	453 (32.4%)	1,464 (35.2%)	
65-74	390 (27.9%)	1,274 (30.7%)	
75-84	331 (23.7%)	1,029 (24.8%)	
85+	224 (16.0%)	387 (9.3%)	
**Gender**			.080
Male	657 (47.0%)	1,841 (44.3%)	
Female	741 (53.0%)	2,313 (55.7%)	
**Education level**			.250
Primary	826 (59.1%)	2,385 (57.4%)	
Secondary	446 (31.9%)	1,230 (29.6%)	
Tertiary	126 (9.0%)	421 (10.2%)	
Missing	0 (0.0%)	118 (2.8%)	
**Activity limitations (mean, SD)**	1.9 (4.1)	1.0 (2.7)	<.001
0	991 (70.8%)	3,266 (78.6%)	
1+	402 (28.8%)	884 (21.3%)	
Missing	5 (0.4%)	4 (0.1%)	
**Chronic diseases (mean, SD)**	2.0 (1.7)	1.9 (1.6)	.054
0	298 (21.3%)	790 (19.0%)	
1	331 (23.7%)	1,145 (27.6%)	
2+	769 (55.0%)	2,215 (53.3%)	
Missing	0 (0.0%)	4 (0.1%)	
**EURO-D (mean, SD)**	2.4 (2.7)	2.4 (2.5)	.690
<4	847 (60.6%)	2,777 (66.9%)	
≥4	312 (22.3%)	1,034 (24.8%)	
Missing	239 (17.1%)	343 (8.3%)	
**Memory test**			.540
Fair/Poor	576 (41.2%)	1,616 (38.9%)	
Excellent/Very good/Good	822 (58.8%)	2,398 (57.7%)	
Missing	0 (0.0%)	140 (3.4%)	
**Hearing test**			.020
Fair/Poor	384 (27.5%)	1,010 (24.3%)	
Excellent/Very good/Good	1,014 (72.5%)	3,139 (75.6%)	
Missing	0 (0.0%)	5 (0.1%)	
**Eyesight**			.003
Fair/Poor	357 (25.5%)	901 (21.7%)	
Excellent/Very good/Good	1,041 (74.5%)	3,245 (78.1%)	
Missing	0 (0.0%)	8 (0.2%)	
**Loneliness (mean, SD)**	3.8 (1.4)	3.7 (1.3)	.100
Low	916 (65.5%)	3,106 (74.8%)	
High	244 (17.5%)	717 (17.2%)	
Missing	238 (17.0%)	331 (8.0%)	
**Social isolation (mean, SD)**	2.3 (0.9)	2.1 (0.9)	<.001
Low	646 (46.2%)	2,496 (60.1%)	
High	482 (34.5%)	1,205 (29.0%)	
Missing	270 (19.3%)	453 (10.9%)	
**Baseline CASP (mean, SD)**	35.9 (6.6)	36.1 (6.3)	.500
Missing	279 (20.0%)	405 (9.7%)	

Note: p-values obtained using ANOVA and Pearson's chi-square test

Supplementary table 3. Baseline characteristics (means, proportions and 95% CI) of unweighted and unimputed sample by country

	**Sweden** n=2995	**Spain** n=4154
**Age (mean, SD)**	70.4 (8.7)	69.9 (10.2)
50-64	805 (26.9%)	1464 (35.2%)
65-74	1272 (42.5%)	1274 (30.7%)
75-84	726 (24.2%)	1029 (24.8%)
85+	192 (6.4%)	387 (9.3%)
**Gender**		
Male	1377 (46.0%)	1841 (44.3%)
Female	1618 (54.0%)	2313 (55.7%)
**Education level**		
Primary	598 (20.0%)	2385 (57.4%)
Secondary	1369 (45.7%)	1230 (29.6%)
Tertiary	967 (32.3%)	421 (10.1%)
Missing	61 (2.0%)	118 (2.8%)
**Activity limitations (mean, SD)**	0.4 (1.4)	1.0 (2.7)
0	2516 (84.0%)	3266 (78.6%)
1+	475 (15.9%)	884 (21.3%)
Missing	4 (0.1%)	4 (0.1%)
**Chronic diseases (mean, SD)**	1.4 (1.4)	1.9 (1.6)
0	878 (29.3%)	790 (19.0%)
1	932 (31.1%)	1145 (27.6%)
2+	1180 (39.4%)	2215 (53.3%)
Missing	5 (0.2%)	4 (0.1%)
**EURO-D (mean, SD)**	1.9 (1.8)	2.4 (2.5)
<4	2438 (81.4%)	2777 (66.9%)
≥4	503 (16.8%)	1034 (24.8%)
Missing	54 (1.8%)	343 (8.3%)
**Memory test**		
Fair/Poor	771 (25.7%)	1616 (38.9%)
Excellent/Very good/Good	2147 (71.7%)	2398 (57.7%)
Missing	77 (2.6%)	140 (3.4%)
**Hearing test**		
Fair/Poor	556 (18.6%)	1010 (24.3%)
Excellent/Very good/Good	2435 (81.3%)	3139 (75.6%)
Missing	4 (0.1%)	5 (0.1%)
**Eyesight**		
Fair/Poor	379 (12.7%)	901 (21.7%)
Excellent/Very good/Good	2612 (87.2%)	3245 (78.1%)
Missing	4 (0.1%)	8 (0.2%)
**Loneliness (mean, SD)**	3.7 (1.1)	3.7 (1.3)
Low	2454 (81.9%)	3106 (74.8%)
High	512 (17.1%)	717 (17.2%)
Missing	29 (1.0%)	331 (8.0%)
**Social isolation (mean, SD)**	1.8 (0.9)	2.1 (0.9)
Low	2241 (74.8%)	2496 (60.1%)
High	588 (19.7%)	1205 (29.0%)
Missing	166 (5.5%)	453 (10.9%)
**Baseline CASP (mean, SD)**	39.6 (5.0)	36.1 (6.3)
Missing	124 (4.1%)	405 (9.7%)
